# Implementation of a Multidisciplinary Transitional Home Care Program for Very-Low-Birth-Weight Infants: A Structured Program Evaluation

**DOI:** 10.3390/healthcare14131919

**Published:** 2026-07-01

**Authors:** Chia-Wen Hung, Li-Min Wu

**Affiliations:** 1 Department of Nursing, Kaohsiung Chang Gung Memorial Hospital, Kaohsiung 833, Taiwan; 2 School of Nursing, Kaohsiung Medical University, Kaohsiung 807, Taiwan; painting@kmu.edu.tw; 3 Department of Medical Research, Kaohsiung Medical University Hospital, Kaohsiung 807, Taiwan

**Keywords:** very low-birth-weight infants, transitional care, continuity of care, multidisciplinary care, telehealth, caregiver stress, integrated care, peer support

## Abstract

**Background:** Very-low-birth-weight (VLBW) infants require ongoing medical follow-up and coordinated family support after discharge due to their immature physiological development and a high risk of complications. Fragmented transitional care and caregiver burden may compromise follow-up adherence and infant health outcomes. This study aimed to describe the implementation, feasibility, and service-level outcomes of a multidisciplinary transitional home care program designed to support continuity of care and family-centered transitional support for high-risk infants through a retrospective descriptive program evaluation. **Methods:** Since 2022, our hospital has implemented a government-supported transitional home care program for low and VLBW infants. A multidisciplinary team provided individualized discharge planning, risk stratification, home-based follow-up, telehealth consultations, developmental monitoring, caregiver education, and psychosocial support. Program outcomes were evaluated using enrollment coverage, follow-up completion, developmental assessment attendance, caregiver stress scores, and service utilization. **Results:** From 2022 to September 2025, enrollment coverage reached 97.7–100% for infants ≤ 1500 g and 100% for preterm infants > 1500 g. A total of 949 video consultations and 2168 telephone or in-person follow-ups were conducted, totaling 3117 service encounters. Developmental assessment attendance rates reached 95%, 93%, and 88% at scheduled corrected-age intervals. Mean caregiver stress scores showed favorable observational trends, decreasing from 14.64 to 10.81. Fifty-two referrals to social resources enhanced service accessibility and family support. **Conclusions:** This multidisciplinary transitional home care program demonstrated high enrollment coverage and sustained follow-up engagement within a tertiary medical center setting. The findings support the feasibility and potential applicability of integrated and family-centered transitional care models in supporting continuity of care and caregiver support for high-risk infants after discharge. Due to the descriptive retrospective design and absence of a control group, causal relationships cannot be established.

## 1. Introduction

Preterm birth and very low birth weight (VLBW) remain major contributors to neonatal morbidity, mortality, and long-term neurodevelopmental impairment worldwide [1,2,3]. Although advances in neonatal intensive care have substantially improved survival, infants born preterm or with VLBW continue to face elevated risks of respiratory disease, feeding difficulties, growth faltering, neurodevelopmental delay, and repeated healthcare utilization after discharge [1,4]. International evidence emphasizes that optimal outcomes for preterm and low-birth-weight infants require not only high-quality inpatient management but also structured post-discharge follow-up and coordinated support across care settings [5,6].

Continuity of care after discharge is particularly challenging for high-risk newborns. Families frequently experience uncertainty regarding symptom recognition, feeding and medication management, and appropriate responses to acute changes [7,8]. Fragmented appointments, transportation barriers, and limited access to timely professional advice may contribute to missed follow-up and delayed care seeking [6,7,8]. In addition to medical vulnerability, parents of very preterm and VLBW infants commonly experience psychological distress, anxiety, and parenting stress, which may affect caregiving capacity and early child development [9,10]. Structured parental support interventions have been associated with improved coping and reduced stress in neonatal populations [11,12,13]. Preterm and very low-birth-weight infants remain medically vulnerable after hospital discharge and are at increased risk of unplanned healthcare utilization and hospital readmission during early infancy. Structured transitional follow-up and continuity-focused care models may help facilitate early identification of medical and developmental concerns while supporting caregiver preparedness after discharge [14].

Transitional care models—including standardized discharge preparation, nurse-led follow-up, and multidisciplinary coordination—have demonstrated benefits in improving care engagement and continuity [7,15]. Educational approaches that incorporate multimedia tools and simulation-based strategies may enhance caregiver competence and confidence in managing complex infant care at home [11,16].

With the increasing integration of telehealth into pediatric practice, remote consultation and digital communication tools have emerged as viable approaches to improve access and responsiveness in post-discharge care [17,18,19]. Telehealth-supported follow-up in neonatal populations has been associated with high caregiver satisfaction and improved accessibility to specialist guidance [19,20]. Furthermore, digital health strategies are attracting increasing recognition as important components of health system strengthening and integrated service delivery [21].

Recent WHO recommendations and international neonatal follow-up guidelines have emphasized the importance of longitudinal developmental surveillance, family-centered transitional care, parental involvement in neonatal intensive care, and early psychosocial support for preterm infants and their caregivers [22,23]. Advances in neonatal medicine, increasing maternal age, assisted reproductive technologies, and improved survival of medically vulnerable infants have further increased the complexity and long-term follow-up needs of preterm populations. Early caregiver–infant interaction, skin-to-skin care, and parental involvement during neonatal intensive care have also been associated with neurodevelopmental and psychosocial outcomes in vulnerable infants [24,25,26]. In addition, the COVID-19 pandemic accelerated the adoption of telehealth-supported transitional care and remote follow-up strategies for medically vulnerable neonatal populations [27].

Since 2022, our hospital has participated in a national home care program for low and very-low-birth-weight (VLBW) infants and established a multidisciplinary transitional home care and proactive follow-up model. This study aimed to describe the implementation, feasibility, and service-level outcomes of a multidisciplinary transitional home care and proactive follow-up model for low- and very low-birth-weight infants within a tertiary medical center. We hypothesized that implementation of this integrated transitional care model would be associated with high follow-up engagement, sustained developmental assessment participation, and favorable caregiver support trends during program participation.

## 2. Materials and Methods

### 2.1. Study Design and Setting

This study was a retrospective descriptive program evaluation conducted as part of routine healthcare quality improvement activities at Kaohsiung Chang Gung Memorial Hospital, a tertiary medical center in Taiwan. The evaluation aimed to describe the implementation processes, service utilization, follow-up adherence, and caregiver support outcomes of a multidisciplinary transitional home care program for very-low-birth-weight infants. The project utilized routinely collected clinical and program monitoring data and was not originally designed as human subjects research.

### 2.2. Multidisciplinary Transitional Care Model

The multidisciplinary transitional home care model was designed to ensure coordinated and continuous care from hospital to home (Table 1). The model consisted of structured discharge preparation, comprehensive needs assessment, risk stratification, individualized care planning, coordinated follow-up scheduling, and interdisciplinary case review meetings.

Eligible participants included low-birth-weight and very low-birth-weight infants discharged from the neonatal intensive care unit or newborn care units of Kaohsiung Chang Gung Memorial Hospital between January 2022 and September 2025 who were offered enrollment in the hospital’s multidisciplinary transitional home care program. Participation in the program was voluntary.

Infants were eligible if they required ongoing developmental follow-up, caregiver education, nutritional support, or coordinated post-discharge transitional care services.

Infants were excluded if they died prior to discharge, were transferred to another institution prior to the completion of discharge planning, or did not participate in the transitional home care follow-up program after discharge. Because this study was conducted as a retrospective descriptive program evaluation using routinely collected service-monitoring data, only infants with available program participation records were included in the analysis.

The care team included neonatologists, nurses, psychologists, social workers, rehabilitation therapists, and dietitians. A designated nurse case coordinator was responsible for care continuity, follow-up scheduling, interdisciplinary communication, and documentation management. Caregiver education and discharge preparation were supported by routine competency-based clinical assessments that were conducted by the care team to evaluate caregivers’ proficiency and readiness in essential infant caregiving tasks prior to discharge.

Follow-up modalities included outpatient visits, structured telephone follow-ups, video consultations, in-person follow-up visits, and secure messaging communication to ensure timely responses to medical and caregiving concerns.

### 2.3. Community Partnership and Peer Support Integration

To enhance integrated and family-centered care, the program incorporated community collaboration and peer support mechanisms (Table 2). The hospital partnered with a national preterm infant foundation and, in 2025, supported the establishment of a Preterm Infant Care Association to expand psychosocial and social resource support.

Caregivers were connected to secure communication platforms (e.g., LINE-based messaging groups), allowing real-time multidisciplinary consultation and peer interaction. These mechanisms facilitated caregiver knowledge exchange, emotional support, and access to social and financial assistance programs.

Educational interventions included multimedia instructional videos, real-time demonstrations, individualized hands-on training, simulated practice sessions, and structured symptom recognition tools to enhance caregiver competence and confidence.

### 2.4. Outcome Measures

Primary program indicators included enrollment rate, follow-up completion rate, attendance at corrected-age developmental assessments, total service encounters, referral utilization, telehealth engagement, and caregiver stress trends. Program coverage was calculated as the proportion of eligible low-birth-weight and very low-birth-weight infants who were enrolled in the multidisciplinary transitional home care program during the evaluation period. Developmental assessment completion rates were calculated based on the number of infants eligible for each corrected-age follow-up interval during the evaluation period. Infants who were unable to complete scheduled follow-up assessments due to relocation, transfer of care, or loss of contact were classified as incomplete follow-up cases for the corresponding assessment interval. The evaluation primarily focused on implementation- and service-level indicators rather than comprehensive clinical effectiveness outcomes.

Caregiver stress was assessed using the standardized caregiver stress assessment form required by the National Health Promotion Administration’s low- and very-low-birth-weight infant home care program in Taiwan. The assessment was conducted within 7 days after hospital discharge and repeated before 3 months post-discharge as part of routine transitional care follow-up procedures.

All indicators were derived from routinely collected program data for service monitoring and quality improvement purposes rather than for research-driven data collection.

### 2.5. Statistical Analysis

Program outcomes were evaluated primarily using implementation- and service-level indicators, including enrollment coverage, follow-up completion, developmental assessment attendance, caregiver stress trends, referral utilization, and telehealth service engagement. Descriptive statistics, including frequencies, percentages, and mean values, were used to summarize program outcomes. Analyses were conducted using Microsoft Excel (Microsoft Corporation, Redmond, WA, USA).

Because this study was conducted as a retrospective descriptive program evaluation using routinely collected service-monitoring data without a comparator group or standardized longitudinal assessment intervals, analyses were primarily limited to descriptive statistics. Inferential statistical testing was not performed due to variability in follow-up timing and the implementation-oriented nature of the evaluation.

### 2.6. Ethical Considerations

This study was conducted as a hospital-based quality improvement and service evaluation initiative using routinely collected and fully de-identified clinical and program-monitoring data. The project did not involve any additional intervention, experimental procedures, or direct interaction with participants for research purposes and was not originally designed as human subjects research.

According to institutional policy, activities classified as quality improvement or service evaluation using retrospective de-identified data are not considered human subjects research and therefore do not require formal Institutional Review Board (IRB) review. Ethical review and approval were waived by the Ethics Committee of the Kaohsiung Chang Gung Memorial Hospital (protocol code 2026001758B, date 10 April 2026).

All procedures were conducted in accordance with the principles of the Declaration of Helsinki.

## 3. Results

### 3.1. Program Coverage and Service Utilization

Enrollment coverage for infants weighing ≤ 1500 g ranged from 97.7% to 100% annually. The small variation reflected a limited number of eligible infants who were not enrolled in the program. For example, in 2022, one family declined participation because they had prior caregiving experience with a previous preterm infant and considered additional transitional home care support unnecessary.

From January 2022 to September 2025, a total of 3117 follow-up encounters were conducted, including 949 video consultations, 1770 telephone follow-ups, and 398 in-person follow-up visits (Table 3). The overall distribution of follow-up modalities demonstrated that 30.4% of encounters were delivered via video consultation, 56.8% through telephone follow-ups, and 12.8% through in-person follow-up visits.

The declining proportion of video consultations over time may partially reflect changes in care delivery patterns during and after the COVID-19 pandemic. During the early implementation phase, video consultations were more frequently used to support remote transitional care and caregiver education. As the program matured, individualized discharge education and caregiving instruction were strengthened during hospitalization, allowing many routine follow-up concerns to be managed through structured telephone follow-ups. In-person visits were primarily reserved for infants requiring developmental assessment or more comprehensive clinical evaluation.

Annual service utilization showed variation in modality distribution. In 2022, 33.7% of encounters were delivered via video consultation. This proportion increased to 45.7% in 2023 but declined to 23.3% in 2024 and 12.9% in 2025 (January–September), reflecting an increasing reliance on telephone and in-person modalities during later phases of program implementation.

Enrollment rates for infants weighing ≤ 1500 g ranged from 97.7% to 100% annually, while enrollment for infants weighing > 1500 g consistently remained at 100%, indicating near-complete program coverage across eligible populations.

### 3.2. Developmental Follow-Up and Psychosocial Outcomes

Developmental follow-up adherence remained high throughout the program period (Table 4). Completion rates were 95% for the first corrected-age assessment (4–10 months), 93% for the second assessment (10–16 months), and 88% for the third assessment (16–21 months), indicating sustained engagement with longitudinal developmental monitoring. Not all infants had reached eligibility for all corrected-age developmental assessment intervals at the time of analysis because enrollment occurred continuously throughout the study period.

Caregiver stress scores decreased from a mean of 14.64 at initial assessment to 10.81 at follow-up. Given the descriptive retrospective program evaluation design and the absence of a comparator group, inferential statistical testing was not performed. These findings should therefore be interpreted as observational trends during program participation rather than evidence of causal program effectiveness.

A total of 52 families were referred to social and community resources, including financial and psychosocial assistance programs, reflecting the program’s integrated care approach.

### 3.3. Educational Intervention Implementation

Educational strategies were actively implemented throughout follow-up encounters, including multimedia video materials, real-time demonstrations, individualized hands-on teaching, and simulated practice sessions. These approaches were tailored to infant clinical conditions and caregiver learning needs.

Repeated exposure to structured educational content and access to ongoing consultation were intended to support caregiver engagement and provide guidance regarding feeding, medication administration, and symptom monitoring. Continuous availability of multidisciplinary guidance through secure communication platforms enabled timely clarification of concerns and reinforced practical caregiving skills.

Although standardized research-based competency outcome measurements were not systematically analyzed, routine competency-based clinical assessments were conducted during discharge preparation as part of standard clinical care.

### 3.4. Caregiver Psychosocial Outcomes and Community Integration

Mean caregiver stress scores decreased from 14.64 at baseline assessment to 10.81 at follow-up (Table 4). Given the descriptive program evaluation design and absence of a comparison group, inferential statistical testing was not performed. Nevertheless, the observed reduction may reflect favorable psychosocial trends during program participation.

A total of 52 referrals to social and financial assistance programs were facilitated through collaboration with community partners. Peer support networks, multidisciplinary consultation, and linkage to external community resources collectively contributed to an integrated support environment for families of preterm and VLBW infants.

Outcome indicators related to developmental follow-up adherence and caregiver stress are summarized in Table 4.

## 4. Discussion

### 4.1. Continuity of Care and Transitional Coordination

Continuity of care is a key determinant of long-term outcomes in preterm and VLBW infants, who remain vulnerable to neurodevelopmental delay and chronic health complications after discharge [1,2,3,28]. Disruptions during the transition from hospital to home have been associated with reduced follow-up adherence and delayed identification of developmental concerns [4,5]. The sustained enrollment coverage observed in this program is notable compared with previously reported follow-up attrition rates in preterm populations [5].

Transitional care programs integrating structured discharge planning and coordinated follow-up have demonstrated improvements in continuity and engagement in high-risk pediatric populations [6,7]. The high enrollment and developmental follow-up completion rates observed in this study suggest that nurse-led coordination and structured case management may help to support sustained post-discharge monitoring.

### 4.2. Educational Innovation and Caregiver Support

Caregiver preparedness at discharge significantly influences post-discharge safety and parental confidence [8,9]. Parents of VLBW infants often report uncertainty regarding feeding techniques, medication administration, and symptom recognition [10]. Evidence indicates that structured parental support programs and multimodal education—including video-based instruction and simulation training—have been associated with improved caregiver preparedness and supportive caregiving practices [11,16,29].

Recent digital and web-based interventions for parents of NICU infants have further demonstrated improvements in caregiver engagement and psychological outcomes [30].

In our program, multimedia instruction, real-time demonstration, and scenario-based teaching were incorporated into follow-up visits, which may help support caregiver preparedness through repeated and experiential learning exposure.

### 4.3. Telehealth and Hybrid Follow-Up Models

Telehealth has emerged as an important strategy to enhance accessibility and continuity of care for medically complex infants [17,18]. Systematic reviews have demonstrated that telemedicine interventions can improve access to pediatric subspecialty services while maintaining high caregiver satisfaction [19]. Telehealth-supported NICU transition programs have shown promise in improving coordination between inpatient and home care settings [31]. The evolution of follow-up modality utilization likely reflected both pandemic-related healthcare adaptations and maturation of the transitional care program. During the COVID-19 pandemic, video consultations provided an important mechanism for maintaining continuity of care while minimizing unnecessary hospital exposure. As the program developed, caregiver education, discharge preparation, and individualized care guidance were increasingly strengthened during hospitalization, reducing reliance on video-based follow-up for routine concerns. Consequently, telephone follow-ups became sufficient for many ongoing support needs, whereas in-person visits were selectively utilized for infants requiring developmental monitoring or more comprehensive clinical evaluation.

The findings of this program evaluation suggest that multidisciplinary transitional care models incorporating structured discharge preparation, coordinated follow-up, telehealth support, caregiver education, and psychosocial referral mechanisms may represent feasible clinical strategies to support continuity-focused neonatal care after discharge. Integration of nurse-led coordination and multimodal communication approaches may also help to facilitate timely caregiver guidance and continuity of developmental monitoring in medically vulnerable infant populations.

Recent developments in remote patient monitoring and mobile-supported care models further highlight the potential of digital platforms to mitigate disparities and enhance responsiveness in neonatal follow-up [32,33]. In this study, telehealth interactions accounted for a substantial proportion of follow-up encounters, supporting a hybrid care model that combines in-person assessments with remote consultations to improve flexibility and accessibility.

### 4.4. Caregiver Stress and Psychosocial Support

Parents of preterm infants are at increased risk of psychological distress, including postpartum depression and parenting stress [34,35]. Family-centered and integrated care approaches have been associated with improved parental coping and reduced stress levels [23,24]. The observed reduction in caregiver stress scores in this study is consistent with prior findings indicating that structured support and coordinated follow-up may positively influence caregiver well-being.

Recent meta-analytic evidence further supports the effectiveness of parenting interventions in reducing parenting stress and improving parent–child interaction outcomes [28,36]. Although causal inference cannot be established due to the absence of a control group, the psychosocial trends observed in this program align with existing literature emphasizing the importance of structured family support in neonatal care.

Emerging evidence regarding epigenetic programming and the importance of the first 1000 days of life further suggests that early caregiver–infant interaction, parental emotional support, and family-centered neonatal care may influence long-term neurodevelopmental and psychosocial outcomes in medically vulnerable infants [23,24,25,26]. Supportive transitional care models that facilitate caregiver engagement, developmental follow-up, and continuity of care may therefore represent important components of early developmental support for preterm populations.

### 4.5. Community Integration and Peer Support

Integrated care frameworks emphasize collaboration between healthcare institutions and community organizations to address medical and social determinants of health [27,37,38]. Peer support programs provide experiential knowledge-sharing and emotional validation, which may strengthen caregiver resilience and empowerment [39]. More recent evidence suggests that structured and virtual peer support initiatives can positively influence NICU family outcomes [40].

The collaboration with a national preterm infant foundation and the establishment of a caregiver association extended support beyond clinical services and reflect an integrated, community-linked model of transitional neonatal care.

Practical lessons from this program may be relevant for healthcare institutions seeking to strengthen continuity-focused neonatal transitional care services. Key implementation elements included early discharge preparation, nurse-led care coordination, multidisciplinary collaboration, telehealth-supported communication, structured caregiver education, and linkage to community and psychosocial support resources. The integration of these components into routine clinical workflows may facilitate sustained follow-up engagement and improve accessibility to transitional care services for medically vulnerable infants and their families. Healthcare systems considering similar programs may adapt these strategies according to local resources, workforce capacity, and healthcare infrastructure.

### 4.6. Limitations

This study was conducted as a retrospective descriptive program evaluation within a single tertiary medical center and did not include a comparator group or formal pre–post analytical framework, thereby limiting causal inference. The reported findings should therefore be interpreted as implementation-related service patterns and observational trends associated with participation in the transitional care program rather than evidence of causal program effectiveness. Outcomes were summarized primarily using descriptive statistics without inferential testing because the evaluation was based on routinely collected implementation and service-monitoring data rather than a prospectively designed comparative study. In addition, comprehensive clinical outcomes, including growth trajectories, hospital readmissions, emergency healthcare utilization, and standardized long-term neurodevelopmental outcomes, were not systematically assessed within the retrospective program-monitoring framework. Standardized research-oriented competency outcome measurements were also not systematically analyzed within the retrospective implementation-monitoring framework. Because this program was implemented within a tertiary referral medical center supported by Taiwan’s national healthcare and public health infrastructure, the transferability of the model to settings with fewer healthcare resources, workforce capacity, or institutional support may require adaptation according to local healthcare system conditions. The present evaluation primarily focused on implementation-related and service-level indicators, including follow-up adherence, developmental assessment attendance, caregiver support trends, referral utilization, and healthcare service engagement. Future research incorporating comparative designs, longitudinal developmental evaluation, multicenter collaboration, and standardized clinical outcome measures is warranted to further examine the implementation, sustainability, and potential effectiveness of multidisciplinary transitional home care models [28].

## 5. Conclusions

This study describes the implementation of a multidisciplinary transitional home care program for low- and very low-birth-weight infants and demonstrates its feasibility within a tertiary healthcare setting. High enrollment coverage, sustained follow-up engagement, and observed caregiver support trends suggest the feasibility and potential value of integrated and family-centered transitional care models in supporting continuity of care for high-risk infants after hospital discharge.

Although causal relationships cannot be established due to the absence of a control group, the findings suggest that integrated and family-centered transitional care models may contribute to continuity-focused support for medically vulnerable infants and their families. The incorporation of multimedia education, simulation-based teaching, secure digital communication, and peer support mechanisms may represent feasible implementation strategies for supporting caregiver engagement and continuity-focused transitional care services.

Future research incorporating comparative designs and long-term developmental outcomes is warranted to further evaluate the effectiveness and sustainability of multidisciplinary transitional home care interventions. From a public health perspective, continuity-focused transitional care programs may contribute to strengthening early developmental surveillance, improving accessibility to coordinated follow-up services, and supporting preventive care strategies for medically vulnerable neonatal populations. Integration of telehealth, caregiver education, psychosocial support, and interdisciplinary coordination may also help reduce barriers to post-discharge care and facilitate earlier identification of developmental or caregiving concerns within the community setting. Nevertheless, this program provides a practical framework for healthcare institutions seeking to improve post-discharge continuity, caregiver support, and integrated neonatal care.

Beyond the observed service outcomes, this program demonstrates a feasible and scalable model for continuity-focused transitional care in medically vulnerable infants. The integration of multidisciplinary coordination, caregiver support, and flexible follow-up strategies may provide valuable guidance for healthcare institutions seeking to strengthen post-discharge care and family-centered neonatal services.

## Figures and Tables

**Table 1:** Structural domains and core components of the multidisciplinary transitional home care model.

Domain	Component	Description
Discharge Preparation	Needs Assessment	Comprehensive assessment of infant clinical status and family needs prior to discharge
	Risk Stratification	Identification of high-risk conditions requiring intensified follow-up
	Individualized Care Plan	Personalized transitional care plan tailored to medical and psychosocial needs
Multidisciplinary Team	Clinical Management	Neonatologist-led medical follow-up
	Nursing Coordination	Designated nurse responsible for care coordination and continuity
	Psychological Support	Assessment and counseling support for caregiver stress
	Social Work Services	Social resource linkage and assistance
	Rehabilitation Services	Developmental monitoring and therapy referral
	Nutritional Support	Feeding assessment and dietary consultation
Follow-Up Modalities	Outpatient Visits	Scheduled corrected-age developmental assessments
	Telephone Follow-Up	Structured follow-up calls for symptom monitoring
	Video Consultation	Remote clinical consultation via secure platform
	Secure Messaging	Ongoing communication through encrypted messaging system
Care Coordination	Case Review Meetings	Interdisciplinary review of complex cases
Community Integration	Foundation Collaboration	Partnership with national preterm infant foundation
	Parent Association	Collaboration with Preterm Infant Care Association
Peer and Social Support	Peer Support Network	Parent-to-parent experiential sharing
	Social Resource Referral	Financial and psychosocial assistance linkage

**Table 2:** Educational, skills-based, and psychosocial support interventions delivered during transitional care.

Intervention Category	Delivery Method	Targeted Outcome
Disease-Specific Instruction	Multimedia instructional videos	Improve caregiver knowledge retention
Real-Time Demonstration	Live video demonstration during follow-up	Enhance procedural understanding
Hands-On Training	Individualized in-person practical sessions	Increase caregiver confidence and competence
Simulated Practice	Scenario-based teaching for feeding/medication administration	Strengthen technical caregiving skills
Symptom Recognition Education	Visual aids and structured checklists	Improve early detection of warning signs
Secure Messaging Consultation	Multidisciplinary team consultation via encrypted platform	Provide timely clinical guidance
Peer Experience Sharing	Parent support group interaction	Reduce emotional stress and enhance coping
Psychosocial Counseling Referral	Social worker or psychologist consultation	Address caregiver stress and mental health needs
Financial Assistance Navigation	Guidance for social and economic support programs	Reduce socioeconomic caregiving burden

**Table 3:** Annual program coverage and service utilization indicators (2022–September 2025).

Year	Total Encounters (n)	Video Consultations (n, %)	Telephone Follow-Ups (n, %)	In-Person Follow-Up Visits (n, %)
2022	1019	343 (33.7%)	560 (55.0%)	116 (11.4%)
2023	692	316 (45.7%)	304 (43.9%)	72 (10.4%)
2024	1043	243 (23.3%)	666 (63.9%)	134 (12.8%)
2025 (Jan–Sep)	363	47 (12.9%)	240 (66.1%)	76 (20.9%)
Total	3117	949 (30.4%)	1770 (56.8%)	398 (12.8%)

**Table 4:** Developmental follow-up adherence and caregiver psychosocial outcomes.

Outcome Measure	Result
Caregiver stress score (mean)	14.64 → 10.81
Developmental assessment completion	
Corrected-age visit 1 (4–10 mo)	148/156 (95%)
Corrected-age visit 2 (10–16 mo)	113/122 (93%)
Corrected-age visit 3 (16–21 mo)	91/103 (88%)
Social resource referrals	52 cases

## Data Availability

The datasets generated and analyzed during the current study are not publicly available due to institutional data protection policies but are available from the corresponding author on reasonable request.

## Abbreviations

VLBW	Very Low Birth Weight.
LBW	Low Birth Weight.
NICU	Neonatal Intensive Care Unit.
LINE	Social communication platform.
